# Predictive Factors of Adequate Bowel Cleansing for Colonoscopy in the Elderly: A Retrospective Analysis of a Prospective Cohort

**DOI:** 10.3390/diagnostics12112867

**Published:** 2022-11-19

**Authors:** Marcello Maida, Antonio Facciorusso, Emanuele Sinagra, Gaetano Morreale, Sandro Sferrazza, Giuseppe Scalisi, Socrate Pallio, Salvatore Camilleri

**Affiliations:** 1 Gastroenterology and Endoscopy Unit, S. Elia-Raimondi Hospital, 93100 Caltanissetta, Italy; 2 Department of Medical and Surgical Sciences, University of Foggia, 71100 Foggia, Italy; 3 Gastroenterology and Endoscopy Unit, Fondazione Istituto San Raffaele Giglio, 90015 Cefalù, Italy; 4 Gastroenterology and Endoscopy Unit, Santa Chiara Hospital, 38100 Trento, Italy; 5 Gastroenterology Unit, ARNAS Garibaldi, 95100 Catania, Italy; 6 Digestive Diseases Endoscopy Unit, Policlinico G. Martino Hospital, University of Messina, 98100 Messina, Italy

**Keywords:** colonoscopy, bowel preparation, PEG, effectiveness, elderly

## Abstract

Factors affecting the quality of bowel preparation for colonoscopy in the elderly are not fully known, and current guidelines provide no specific recommendations. This study aimed to assess the difference in bowel cleansing in young and elderly patients and evaluate predictors of bowel cleansing in the elderly. We retrospectively reviewed a prospective cohort of 1289 patients performing colonoscopy after a 1-, 2-, or 4-L PEG-based preparation. All 1289 were included in the analysis. Overall, 44.6% of patients were aged ≥65 years. Cleansing success (CS) was achieved in 77.3% and 70.3% of patients aged <65 years and ≥65 years, respectively. At multivariable analysis, split regimen (OR = 2.43, 95% CI = 1.34–4.38; *p* = 0.003), adequate cleansing at previous colonoscopy (OR = 2.29, 95% CI = 1.14–4.73; *p* = 0.02), tolerability score (OR = 1.29, 95% CI = 1.16–1.44; *p* < 0.001), a low-fiber diet for at least 3 days (OR = 2.45, 95% CI = 1.42–4.24; *p* = 0.001), and colonoscopy within 5 h after the end of preparation (OR = 2.67, 95% CI = 1.28–5.56; *p* = 0.008) were independently associated with CS in the elderly. Combining a low-fiber diet for at least 3 days, split preparation, and colonoscopy within 5 h allowed a CS rate above 90% and should always be encouraged. A 1-L PEG-ASC preparation was also associated with greater high-quality cleansing of the right colon and may be preferred.

## 1. Introduction

Screening for colorectal cancer (CRC) is highly important since early detection of this neoplasm is associated with improved survival. Colonoscopy is one of the most widely adopted methods for CRC screening, allowing a long-term reduction of disease incidence and mortality [1,2].

Despite this, a quality colonoscopy strictly depends on adequate bowel cleansing, which may affect the diagnostic accuracy and detection of adenomas [3]. Moreover, high quality over adequate cleansing allows for a further improvement of both the adenoma detection rate (ADR) [4] and the detection of sessile serrated polyps [5]. Inadequate preparation results in reduced colonoscopy sensitivity, increased procedural time, greater risk of adverse events, more rescheduled procedures, and higher costs [6,7,8,9].

Several factors have been found to affect the quality of bowel cleansing, including the type of bowel preparation, split-dose regimen, low-fiber diet, comorbidities, concomitant medications, inpatient status, and elderly age [10]. Of these, age is among the factors with the most significant impact since it is a non-modifiable variable. Most patients undergoing screening and surveillance are over 50 years old, and many are elderly [11].

Therefore, identifying the best strategies to improve the quality of preparation in the elderly by ensuring adequate preparation is crucial. However, factors affecting bowel cleansing in the elderly are not fully known, and current guidelines do not provide specific recommendations for these patients [12]. Moreover, the effectiveness and safety profile of the most recent very-low-volume 1-L polyethylene glycol plus ascorbate (PEG-ASC; Plenvu; Norgine, Harefield, UK) preparation in the elderly is not fully known.

This study aimed to assess the difference in bowel cleansing in young and elderly patients and to evaluate predictors of bowel cleansing in the elderly.

## 2. Materials and Methods

### 2.1. Study Design and Participants

This was a retrospective analysis of prospectively collected data. The database used for the analysis was derived from a prospective, multicenter, observational study performed across five Italian gastroenterology and endoscopy units to compare the effectiveness of 1-, 2-, and 4-L PEG-based preparations [13]. That study consecutively enrolled all men and women, in- and outpatients aged >18 years, undergoing a screening, surveillance, or diagnostic colonoscopy, after an afternoon-only or afternoon–morning (split) preparation with 4-L, 2-L, or 1-L PEG.

At the time of colonoscopy scheduling, each patient was provided with a form containing the names of the proposed solutions (1-L, 2-L, and 4-L PEG) and, for each, separate instructions for bowel preparation. The solution was then independently chosen by the patient based on personal preference, costs, and local availability.

### 2.2. Techniques

The split bowel preparation was self-administered, with the first dose taken the afternoon before the colonoscopy at 6.00 PM ± 2 h and the second dose at 5.00 AM ± 2 h the following morning. The solution was prepared with 500 mL of additional clear fluids after each dose, and additional clear fluids ad libitum were permitted up to 2 h before the procedure. A low-fiber diet was recommended for at least 1 day before the colonoscopy, and on the day before the colonoscopy, patients were permitted a light breakfast and lunch.

### 2.3. Outcomes and Measurement

The primary endpoints of the study were the assessment of cleansing success (CS) and high-quality cleansing (HQC) of the right colon. The secondary endpoints were the evaluation of predictors of CS and HQC of the right colon in the elderly.

Demographic, clinical, and anthropometric data were collected at baseline. The elderly population was defined by age ≥65 years. The effectiveness of the preparation was evaluated on the grade of bowel cleansing, which was assessed through the Boston Bowel Preparation Scale (BBPS) [14] by site unblinded colonoscopists after specific training. CS was defined as a total BBPS ≥6 with a partial BBPS ≥2 in each colon segment and an HQC of the right colon as a partial BBPS = 3.

Adherence was defined as the consumption of at least 75% of each dose. Tolerability was evaluated through a semi-quantitative scale with a score ranging from 1 to 10 (1 = lowest rank; 10 = highest rank). Safety was assessed through adverse events (AEs) by patient reporting at the time of colonoscopy and by patient monitoring in the 2 h of observation following the endoscopy. Data on previous colonoscopies were collected by direct viewing of endoscopic reports or, in the absence of these, through the administrative registry.

### 2.4. Statistical Analysis

Continuous variables were reported as mean ± standard deviation, and categoric variables were summarized as frequency and percentage. Comparisons of variables were made by t-test and χ^2^ test as appropriate. Logistic regression models were performed to assess the presence of variables associated with the CS and HQC of the right colon. All statistical analyses were performed using SPSS v. 28.0 for Macintosh (SPSS Inc., Chicago, IL, USA).

### 2.5. Ethics

The study received ethics committee approval and was conducted in accordance with the principles of the Declaration of Helsinki and good clinical practice. Patients provided written informed consent.

## 3. Results

### 3.1. Study Population and Characteristics

A total of 1289 patients were included in the analysis. Of these, 714 (55.2%) were aged <65 years, and 575 (44.8%) were aged ≥65 years. In the elderly group, the mean age was 72.9 ± 5.9 years, 54.6% of patients were males, and 94.3% were outpatients (Table 1). Elderly patients had a higher prevalence of hypertension (56.0% vs. 23.6%, *p* < 0.001), diabetes (19.1% vs. 4.8%, *p* < 0.001), obesity (19.7% vs. 17.2, *p* = 0.252), and constipation (19.8% vs. 14.1, *p* = 0.006). The main indication for colonoscopy was screening or surveillance for CRC in both elderly and non-elderly patients (56.8% and 58.7%, respectively).

Adherence to a low-fiber diet for at least 3 days was similar in elderly and non-elderly patients (85.5% and 86.3%, *p* = 0.696), as was adherence to the bowel preparation (91.4% and 91.4%, *p* = 0.951). The rate of inadequate cleansing at previous colonoscopy was higher in the elderly compared to non-elderly patients (7.0% and 3.8%, *p* = 0.012).

In the elderly group, 39.8%, 42.6%, and 17.6% of patients performed a 4-L, 2-L, and 1-L PEG, respectively, and 38.0% of them were in a split regimen. Among non-elderly patients, 36.6%, 44.9%, and 18.5% performed a 4-L, 2-L, and 1-L PEG, respectively, and 37.3% of them were in a split regimen fashion. A higher tolerability score was registered for 1-L PEG compared to 2-L and 4-L PEG in both elderly (7.7 vs. 7.2 and 7.2, *p* = 0.099) and non-elderly patients (7.8 vs. 7.0 and 7.2, respectively, *p* < 0.001).

### 3.2. Bowel Cleansing Efficacy

**Figure 1:**
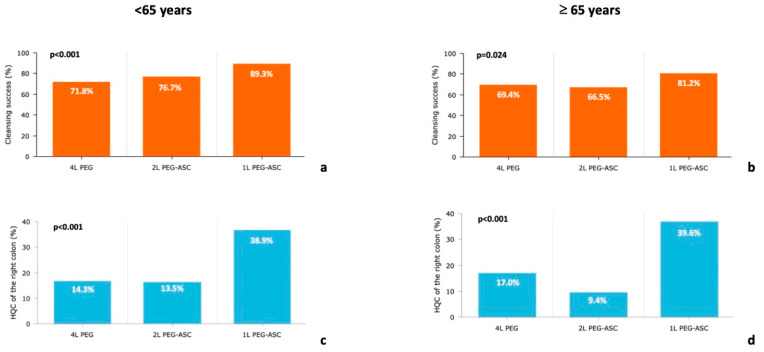
Cleansing success rate by type of bowel preparation in patients <65 years (**a**) and ≥65 years (**b**), and high-quality cleansing of the right colon in patients <65 years (**c**) and ≥65 years (**d**).

Overall, bowel cleansing by BBPS was 6.5 ± 1.5, the CS was 77.3%, and the HQC of the right colon was 18.5%. The CS was lower in patients aged ≥65 years compared to those aged <65 years: 70.3 vs. 77.3% overall (*p* = 0.004), 61.2% vs. 69.5% (*p* = 0.014) in the subgroup of day-before preparation, and 84.9% vs. 90.2% (*p* = 0.078) in the subgroup of split preparation (Figure 1; Table 2).

The HQC of the right colon was similar in patients aged ≥65 years compared to those aged <65 years: 17.7% vs. 18.5% overall (*p* = 0.724), 8.7% vs. 10.8% (*p* = 0.317) in the subgroup of day-before preparation, and 32.6% vs. 31.1% (*p* = 0.723) in the subgroup of split preparation (Figure 1).

When analyzing the effectiveness of bowel cleansing by different PEG volumes in younger patients, CS was achieved in 89.3%, 76.7%, and 71.8% (*p* < 0.001), and HQC of the right colon in 38.9%, 13.5%, and 14.3% (*p* < 0.001), for 1-L, 2-L, and 4-L PEG, respectively.

In elderly patients, CS was achieved in 81.2%, 66.5%, and 69.4% (*p* = 0.024), and HQC of the right colon in 39.6%, 9.4%, and 17.0% (*p* < 0.001), for 1-L, 2-L, and 4-L PEG, respectively.

### 3.3. Predictors of Bowel Cleansing in the Elderly

The logistic multiple regression model for overall CS showed that split regimen (OR = 2.43, 95% CI = 1.34–4.38; *p* = 0.003), adequate cleansing at previous colonoscopy (OR = 2.29, 95% CI = 1.14–4.73; *p* = 0.02), tolerability score (OR = 1.29, 95% CI = 1.16–1.44; *p* < 0.001), low-fiber diet for at least 3 days preceding colonoscopy (OR = 2.45, 95% CI = 1.42–4.24; *p* = 0.001), and colonoscopy within 5 h after preparation (OR = 2.67, 95% CI = 1.28–5.56; *p* = 0.008) were independently associated with CS in the elderly (Table 3).

**Figure 2:**
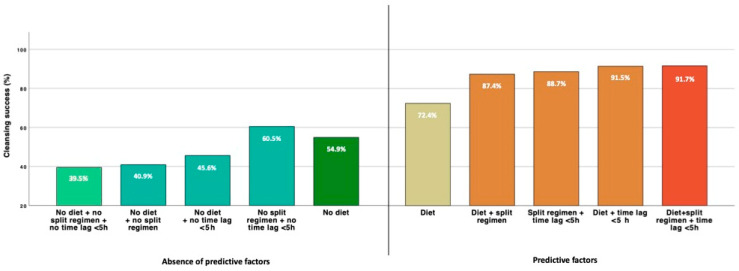
Cleansing success rates in patients ≥ 65 years by combination of different predictive factors.

In patients aged ≥65 years, the combination of at least two variables, including low-fiber diet, split regimen, and colonoscopy within 5 h after preparation, was associated with CS rates ranging between 87.4% and 91.7%. Conversely, the absence of at least two predictors, including a low-fiber diet, dropped the rate below 45.6% (Figure 2).

The logistic multiple regression model for HQC of the right colon showed that preparation with 1-L PEG-ASC over 2-L PEG (OR = 2.77, 95% CI = 1.52–5.03; *p* = 0.001), preparation with 4-L PEG over 2-L PEG (OR = 3.78, 95% CI = 2.03–7.03; *p* < 0.001), split regimen (OR = 4.58, 95% CI = 2.43–8.64; *p* < 0.001), and tolerability score (OR = 1.20, 95% CI = 1.05–1.38; *p* = 0.005) were independently associated with HQC of the right colon in the elderly (Table 3).

### 3.4. Safety

Mild and moderate AEs were reported in 16.8% of patients overall. The number of patients with AEs was lower in the elderly compared to the non-elderly group: 16.0% vs. 20.3% (*p* = 0.046).

The distribution of AEs among different preparation groups was similar in both the elderly (18.3% in 4-L PEG, 13.5% in 2-L PEG, 15.8% in 1-L PEG-ASC, *p* = 0.348) and younger patients (22.8% in 4-L PEG, 18.2% in 2-L PEG, 19.8% in 1-L PEG-ASC, *p* = 0.399).

The most frequent AE was nausea, reported in 5.7% of patients (4.9% in the elderly vs. 6.4% in non-elderly, *p* = 0.253), followed by vomiting in 3.7% (3.7% in the elderly vs. 3.8% in non-elderly, *p* = 0.591), abdominal pain in 2.0% (1.4% in the elderly vs. 2.5% in non-elderly, *p* = 0.146), and thirst in 0.7% (0.2% in the elderly vs. 1.1% in non-elderly, *p* = 0.041; Supplementary Table S1). No severe or serious AEs were reported, and no deaths occurred in either group.

## 4. Discussion

Bowel cleansing in the elderly is challenging and influenced by many variables [15]. Age is a risk factor per se for poor bowel cleansing, and older age is also associated with a higher prevalence of other risk factors, including the presence of comorbidities (e.g., diabetes, hypertension, constipation), concomitant medications (e.g., calcium channel blockers, narcotics, tricyclic antidepressants), or hospitalization [16].

On the other hand, the same population is also the most in need of quality colonoscopy since they are more exposed to screening and surveillance for CRC. Moreover, the increase in life expectancy is leading to an ever-increasing number of diagnostic colonoscopies in the elderly, making the problem more relevant.

However, factors affecting bowel cleansing in the elderly are not fully known, and current guidelines do not provide specific recommendations to optimize bowel preparation in these patients.

The results from this study confirm that elderly patients achieved lower rates of CS overall, both in the afternoon–morning and split regimen subgroups, with a similar HQC. Among all variables, split regimen, adequate cleansing at previous colonoscopy, tolerability score, low-fiber diet for at least 3 days preceding colonoscopy, and colonoscopy within 5 h after preparation were independently associated with CS in the elderly.

Some of these factors, such as preparation regimen and timing of colonoscopy after preparation, are already well-known risk factors for inadequate bowel cleansing in all age groups.

We also found that a low-fiber diet for at least 3 days preceding colonoscopy was independently associated with CS in the elderly. This is in contrast to current guidelines, which recommend a low-fiber diet only on the day preceding the colonoscopy [12]. However, this recommendation is mostly derived from studies conducted in regions of the world where fiber consumption is inherently low. In regions where fiber consumption is higher, such as the one from which this study was derived, a prolonged low-fiber diet is probably associated with greater efficacy.

Higher tolerability of bowel preparation is associated with better cleansing since a well-tolerated product is more likely to be associated with greater adherence and preparation completeness.

Considering only modifiable variables affecting bowel cleansing in the elderly, the strategy of combining at least two, including a low-fiber diet, split regimen, and timing of colonoscopy within 5 h after preparation, may contribute to achieving a significantly higher rate of CS between 87.4% and 91.7%. All these factors are simple and easy to achieve but imply proper patient education. For very elderly patients, information may not be sufficient, and careful assistance from family members or carers may be necessary.

Moreover, performing the colonoscopy within 5 h from the end of the preparation depends on not only the patient but also the adequate organization of the endoscopy unit, which must guarantee the execution of the procedure within the expected times.

We found that split regimen, tolerability score, and 1-L and 4-L PEG solutions over 2-L PEG were independently associated with HQC of the right colon in the elderly. The higher effectiveness of 1-L PEG-ASC is consistent with previous data showing its higher effectiveness compared to other PEG preparations [13,17,18,19,20,21,22,23,24]. This element is also significant since choosing a more performant solution may be helpful to maximize the outcome in this difficult-to-treat population. In this line, a recent randomized controlled trial comparing 1-L PEG-ASC with 2-L PEG-ASC in elderly patients undergoing colonoscopy showed a similar CS between the two solutions, with a higher rate of perfect cleansing and HQC of the right colon with 1-L PEG-ASC, with a comparable incidence of AEs [25]. Moreover, the lower volume of the 1-L PEG-ASC may be easier to consume, with higher tolerability compared to other solutions. This is relevant since, in our population, tolerability has been found to be an independent predictor of both CS and HQC of the right colon.

We found a similar safety profile in both the elderly and non-elderly populations, without a significant difference in the incidence of total AEs or the AE types, except for thirst, which was more frequent in younger patients. AEs were mild to moderate and not able to affect the completeness of the bowel preparation, with adherence achieved in more than 90% of patients in both groups. Finally, the overall rate of AEs did not differ according to the preparation type used in the elderly or younger patients. This is a key element since some concerns have been raised regarding the safety of 1-L PEG-ASC preparation in the elderly. In this regard, none of the three pivotal randomized controlled trials identified differences in AE rates according to age [17,18,19]. Similarly, in this study, we did not identify variation in AE incidence by the type of preparation consumed.

This study has several strengths. It was performed on a large sample size of elderly patients in a real-life setting, comparing different volumes of PEG preparations. However, we should acknowledge some limitations. First, even though the patients were enrolled prospectively, the analysis was retrospective, exposing it to the potential risk of bias. Second, the absence of blinding between site colonoscopists and the type of bowel preparation is another drawback. Third, despite the indication of the participating centers, adherence to the split regimen was suboptimal. This may underestimate bowel preparation since a split regimen is an established factor associated with higher cleansing efficacy.

In conclusion, the results from this study highlight the difficulty in obtaining quality preparation in the elderly and identify predictive factors for adequate bowel cleansing. In these patients, the combination of a low-fiber diet for at least 3 days before the colonoscopy, a split preparation regimen, and a colonoscopy performed within 5 h after the end of preparation allowed a CS rate above 90% and should always be encouraged. 1-L PEG-ASC preparation was also associated with greater HQC of the right colon and may be preferred when feasible.

Of course, these results must be confirmed by further prospective and controlled studies evaluating the effectiveness of different preparation regimens and solutions in the elderly population.

## Figures and Tables

**Table 1:** Baseline patients’ characteristics.

	<65 Years (N = 714)	≥65 Years (N = 575)	*p*
Male sex, n (%)	363 (51.3%)	314 (54.6%)	0.234
Age, years, mean (SD)	50.5 ± 10.5	72.9 ± 5.9	<0.001
Weight, kg, mean (SD)	72.7 ± 15.2	71.9 ± 13.8	0.335
Height, cm, mean (SD)	167.4 ± 9.2	163.8 ± 8.4	<0.001
BMI, mean (SD)	25.8 ± 4.6	26.8 ± 4.7	<0.001
Comorbidities			
- Hypertension	167 (23.6%)	322 (56.0%)	<0.001
- Diabetes	34 (4.8%)	110 (19.1%)	<0.001
- Constipation	100 (14.1%)	114 (19.8%)	0.006
- Obesity	122 (17.2%)	113 (19.7%)	0.252
Colonoscopy indication, n (%)			
- Screening	279 (39.1%)	164 (28.5%)	
- Surveillance	140 (19.6%)	163 (28.3%)	<0.001
- Diagnostic	295 (41.3%)	248 (43.2%)	
Previous inadequate cleansing	27 (3.8%)	40 (7.0%)	0.012
Outpatients	692 (97.7%)	542 (94.3%)	0.001
Low fiber diet ≥ 3 days	605 (86.3%)	845 (85.5%)	0.696
Split preparation regimen	264 (37.3%)	218 (38.0%)	0.815
Type of bowel solution			
- 4L PEG	259 (36.6%)	229 (39.8%)	
- 2L PEG-PEG/ASC	318 (44.9%)	245 (42.6%)	0.492
- 1L PEG/ASC	131 (18.5%)	101 (17.6%)	
Adherence to bowel preparation	653 (91.4%)	526 (91.4%)	0.951

**Table 2:** Outcomes of bowel cleansing in younger and elderly patients.

	<65 Years (N = 714)	≥65 Years (N = 575)	*p*
Cecal intubation rate	97.5%	95.5%	0.05
BBPS total, mean (SD)	6.6 ± 1.4	6.3 ± 1.6	0.007
- Left colon, mean (SD)	2.2 ± 0.5	2.2 ± 0.6	0.872
- Transverse colon, mean (SD)	2.1 ± 0.6	2.0 ± 0.6	0.166
- Right colon, mean (SD)	1.7 ± 0.6	1.7 ± 0.7	0.986
Bowel cleansing success by preparation regimen			
- Overall	77.3%	70.3%	0.004
- Day before regimen	69.5%	61.2%	0.014
- Afternoon/morning regimen	90.2%	84.9%	0.078
Bowel cleansing success by type of solution			
- 4L PEG	71.8%	69.4%	0.564
- 2L PEG	76.7%	66.5%	0.007
- 1L PEG	89.3%	81.2%	0.079
HQC of the right colon by preparation regimen			
- Overall	18.5%	17.7%	0.724
- Day before regimen	10.8%	8.7%	0.317
- Afternoon/morning regimen	31.1%	32.6%	0.723
HQC of the right colon by type of solution			
- 4L PEG	14.3%	17.0%	0.404
- 2L PEG	13.5%	9.4%	0.131
- 1L PEG	38.9%	39.6%	0.917
Tolerability by type of solution			
- 4L PEG	7.2 ± 1.9	7.4 ± 1.9	0.466
- 2L PEG	7.0 ± 1.9	7.2 ± 1.9	0.224
- 1L PEG	7.8 ± 1.9	7.7 ± 1.9	0.595

**Table 3:** Logistics multiple regression model estimates for overall cleansing success and high-quality cleansing of the right colon in the elderly.

Predictors	Estimates	95% CI	*p*
**Cleansing success**			
Split regimen	2.43	1.34–4.38	0.003
Adequate cleansing at previous colonoscopy	2.29	1.14–4.73	0.002
Tolerability Score	1.29	1.16–1.44	<0.001
Low fiber diet ≥ 3 days	2.45	1.42–4.24	0.001
Colonoscopy within 5 h after preparation	2.67	1.28–5.56	0.008
**High-quality cleansing of the right colon**			
Solution type (ref = 2L PEG)			
1L PEG-ASC	2.77	1.52–5.03	0.001
4L PEG	3.78	2.03–7.03	<0.001
Split regimen	4.58	2.43–8.64	<0.001
Adherence > 75%	2.89	0.35–23.5	0.321
Colonoscopy within 5 h after preparation	0.99	0.55–1.79	0.986
Tolerability Score	1.20	1.05–1.38	0.005

## Supplementary Materials

The following supporting information can be downloaded at: https://www.mdpi.com/article/10.3390/diagnostics12112867/s1, Table S1: Incidence of adverse events in younger and elderly patients.
