# Supplementary material for: Predictive Factors of Adequate Bowel Cleansing for Colonoscopy in the Elderly: A Retrospective Analysis of a Prospective Cohort
Source: Diagnostics (Basel). 2022 Nov 19;12(11):2867. doi: 10.3390/diagnostics12112867 (PMC9689943; doi:10.3390/diagnostics12112867)
Supplement: Supplementary file 1 [file diagnostics-12-02867-s001.zip › diagnostics-2002199-supplementary.pdf]

diagnostics

Supplementary Table S1. Incidence of adverse events (AEs) in younger and elderly patients.

	**<65 Years (N = 714)**	**≥65 Years (N = 575)**	***p***
Patients with AEs, n (%)	144 (20.3%)	92 (16.0)	0.046
Nausea, n (%)	45 (6.4%)	28 (4.9%)	0.253
Vomiting, n (%)	27 (3.8%)	21 (3.7%)	0.591
Abdominal pain, n (%)	18 (2.5%)	8 (1.4%)	0.146
Thirst, n (%)	8 (1.1%)	1 (0.2%)	0.041
Patients with serious AEs, n (%)	0	0	-
